# As a carrier–transporter for hair follicle reconstitution, platelet-rich plasma promotes proliferation and induction of mouse dermal papilla cells

**DOI:** 10.1038/s41598-017-01105-8

**Published:** 2017-04-25

**Authors:** Shun-E. Xiao, Yong Miao, Jin Wang, Wei Jiang, Zhe-Xiang Fan, Xiao-Min Liu, Zhi-Qi Hu

**Affiliations:** Department of Plastic and Aesthetic Surgery, Nan Fang Hospital, Southern Medical University, Guangzhou, Guangdong 510515 China

## Abstract

Morphogenesis of hair follicles during development and in hair reconstitution assays involves complex interactions between epithelial cells and dermal papilla cells (DPCs). DPCs may be a source of cells for hair regeneration in alopecia patients. Reconstitution of engineered hair follicles requires *in vitro* culture of trichogenic cells, a three-dimensional scaffolds, and biomolecular signals. However, DPCs tend to lose their biological activity when cultured as trichogenic cells, and scaffolds currently used for hair follicle regeneration lack biological efficiency and biocompatibility. Platelet-rich plasma (PRP) gel forms a three-dimensional scaffold that can release endogenous growth factors, is mitogenic for a variety of cell types and is used in model tissue repair and regeneration systems. We found that 5% activated PRP significantly enhanced cell proliferation and hair-inductive capability of mouse and human DPCs *in vitro* and promoted mouse hair follicle formation *in vivo*. PRP also formed a three-dimensional gel after activation. We used PRP gel as a scaffold to form many de novo hair follicles on a plane surface, showing it to be candidate bioactive scaffold capable of releasing endogenous growth factors for cell-based hair follicle regeneration.

## Introduction

Alopecia is a common disorder that can cause significant psychological stress and affect self-confidence, which in turn leads to a desire of therapeutic treatment^1^. Drugs or autologous hair follicle transplantation are the primary treatments of alopecia. However, drug treatment only delays hair loss. Transplantation only redistributes the remaining hair follicles and thousands usually need to be moved to achieve a cosmetically favourable appearance. The effectiveness of both therapeutic options are limited because new hair follicles cannot be regenerated. In some severe cases of hair loss, in which enough hair follicles cannot be obtained, hair follicle regeneration through bioengineering is a promising alternative^2, 3^.

Engineered hair follicle reconstitution methods currently require a combination of trichogenic cells, three-dimensional scaffolds, and biomolecular signals^4^. The dermal papilla (DP), a cluster of specialized fibroblasts located in hair follicles, is believed to have a key role in hair follicle morphogenesis and cycling^5^. Dermal papilla cells (DPCs) participate in hair reconstitution as intact tissue, freshly dissociated cell preparations, or cultured cells, and serve as the source of cells for hair regeneration^6–8^. *In vitro* regeneration of hair follicles requires many cultured DPCs, but DPCs gradually lose hair-inductive capacity during subculture^9, 10^. Many strategies are used to maintain the hair-inductivity of cultured DPCs^11–13^. However, it is difficult to obtain large numbers of DPCs with inductive capacity under current culture conditions in a short time. There is a need of improved culture conditions for DPCs.

Various natural and synthetic biomaterials have been used as scaffolds for hair follicle regeneration^14–16^, but their biological efficiency and biocompatibility are not stable. Scaffolds for hair follicle regeneration that have excellent biodegradability, biocompatibility, and cost-effectiveness are needed. Endogenous human skin equivalent provides a physiological environment which could generate follicle-like structure *in vitro*, highlighting the great role of the dermal extra cellular matrix (ECM) in controlling the hair follicle-like structure formation *in vitro* ^17^. In fact, there is a growing awareness of the fact that ECM plays multiple roles in cell growth, survival, differentiation and morphogenesis^18^. Autologous biomaterial scaffolds obtained from individual patients have long been an attractive option. Platelet-rich plasma (PRP), has been used in tissue repair and regeneration and is a treatment option in clinical practice^19–22^. PRP is used as an innovative therapy for hair restoration, which has become increasingly common. Use of PRP in hair restoration has been investigated for many conditions including androgenetic alopecia and alopecia areata^23, 24^. PRP contains a high concentration of thrombocytes that release numerous growth factors, including platelet derived growth factor (PDGF), fibroblast growth factor (FGF), and vascular endothelial growth factor, when activated. Thrombocyte activation can thus enhance recruitment, proliferation, and differentiation of cells for tissue regeneration^25–27^. FGF2 and PDGF are potent mitogens for cells of mesenchymal origin. Previous studies have shown that FGF2 both enhances the proliferation and maintains the hair-inductive capacity of DPCs^28^. PDGF is essential for the induction and maintenance of anagen-phase hair follicles *in vivo* ^29^, and FGF2 and PDGF have recently been shown to synergistically enhance cell proliferation and the hair-inductive activity of DPCs^30^. PRP can be activated by thrombin or calcium to form fibrin gels that have been used as a three-dimensional scaffolds capable of releasing endogenous growth factors for cartilage tissue engineering^31^. In this study, we tested the efficacy of PRP to promote cell proliferation and hair-inductive activity in human scalp DPCs and murine vibrissal DPCs, and evaluated endogenous growth factor release and hair follicle reconstitution using PRP bioactive scaffolds.

## Results

### Platelet count and growth factor levels

Mean platelet counts in whole blood and PRP were 18.80 × 10^4^/μl and 133.5 × 10^4^/μl respectively. The concentration of platelets in PRP was approximately 7.1 times greater than that in whole blood. The mean concentrations of PDGF-AB and FGF2 in supernatants of activated PRP were 239.17 ± 17.74 ng/ml, 247.37 ± 14.28 ng/ml respectively.

### Effect of activated PRP on human and mouse DPC proliferation and hair-inductive activity

**Figure 1:**
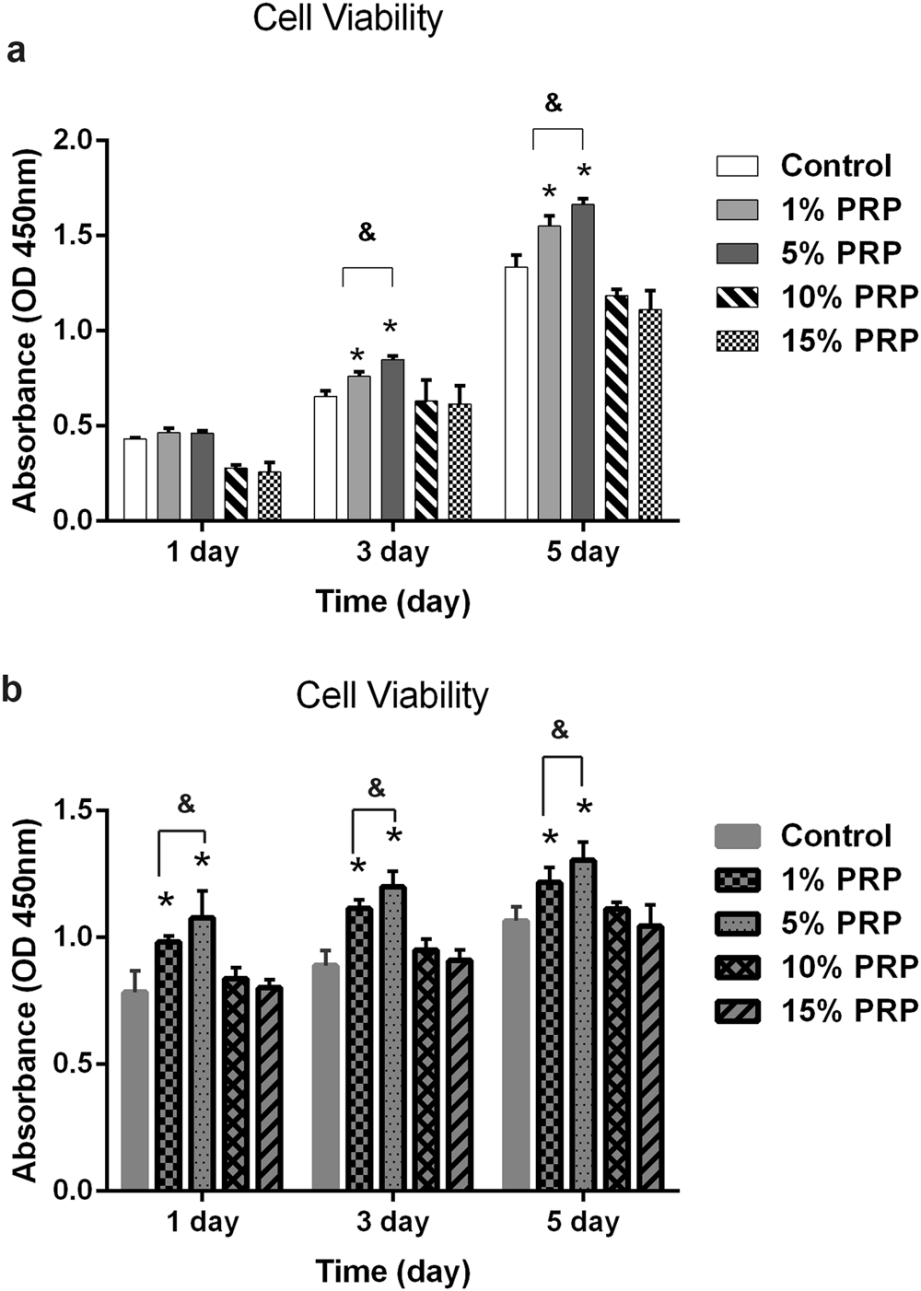
Proliferation of mouse dermal papilla cells (DPCs) (**a**) and human dermal papilla cells (**b**) on days 1, 3, and 5 after addition of various concentrations of activated platelet-rich plasma (PRP). The addition of 1% and 5% activated PRP to the culture medium promoted cell proliferation. Maximal promotion occurred with 5% PRP; 10% or 15% PRP did not promote further. *p < 0.05 vs. control and ^&^p < 0.05 vs. 1% PRP.

**Figure 2:**
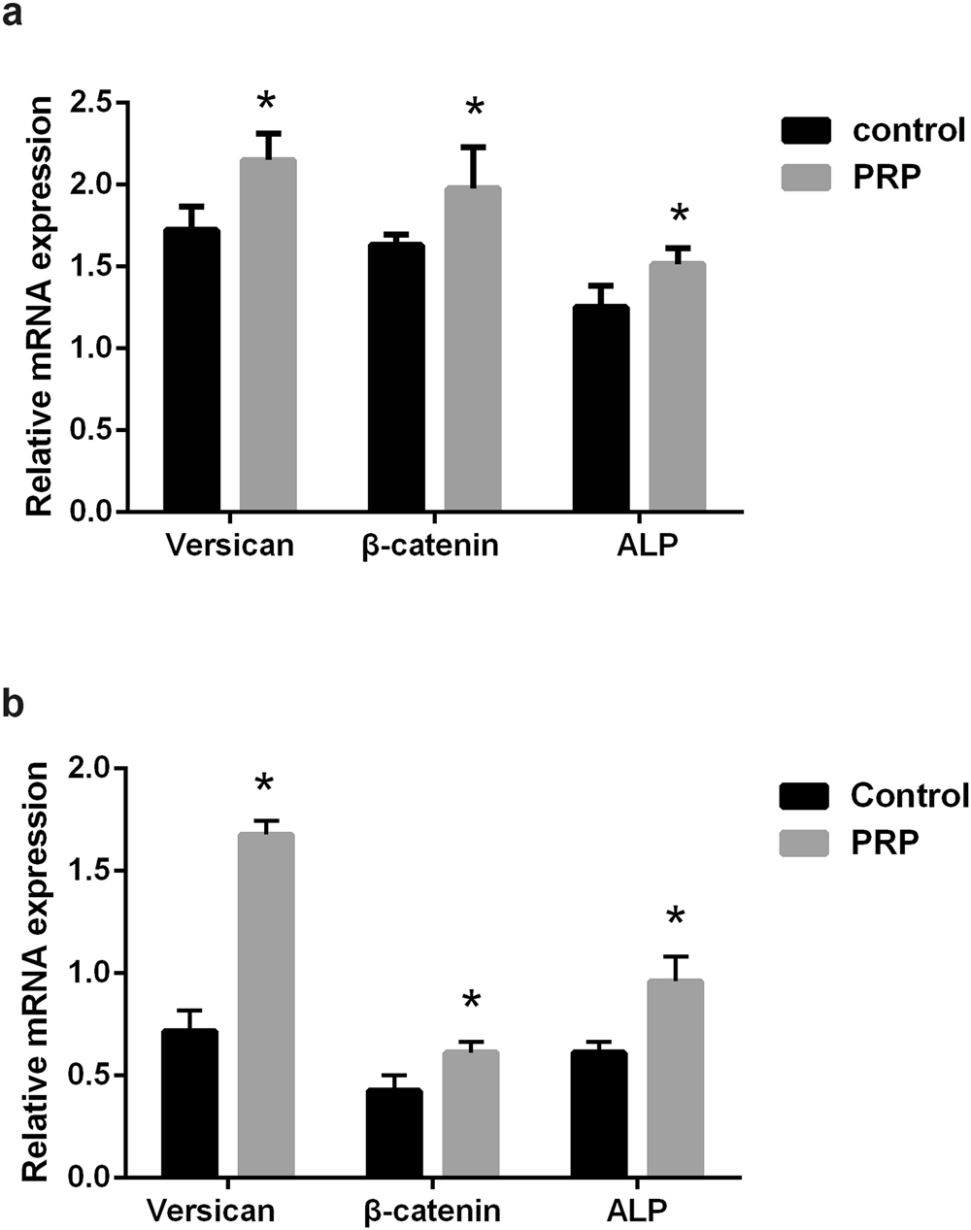
Quantitative real-time PCR (qPCR) assay of P3 DPCs cultured in general medium (control) or medium with addition of 5% activated PRP (PRP). (**a**) Mouse marker genes of DP anagen were analysed (n = 3). (**b**) Human marker genes of DP anagen were analysed (n = 3). *p < 0.05 vs. control.

**Figure 3:**
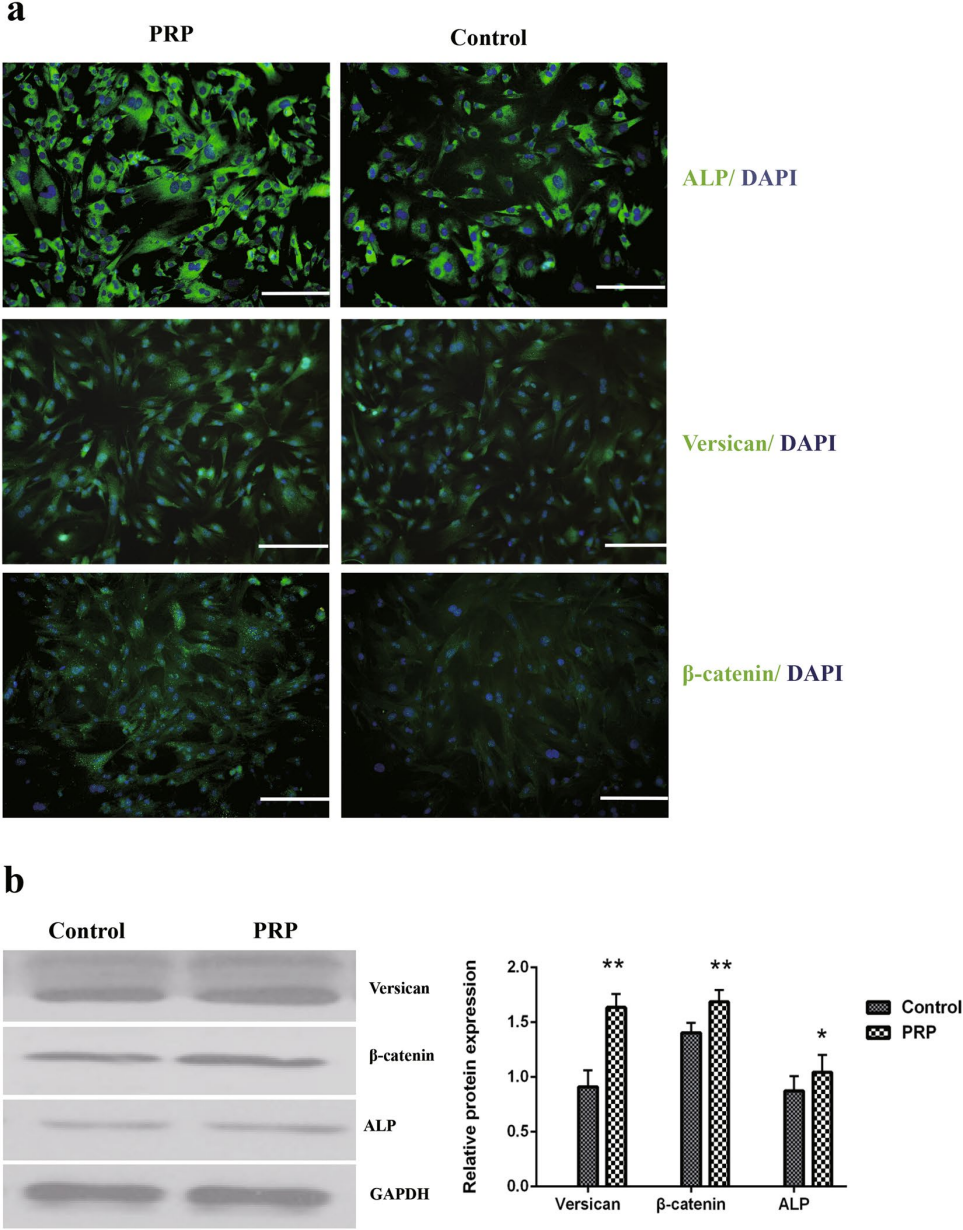
DPC marker proteins of mouse were assayed by immunofluorescence staining and immunoblotting. (**a**) Consistent with qPCR, immunofluorescent images show increased protein expression in PRP-treated DPCs compared with controls. Scale bars = 200 μm. (**b**) Western blot analysis quantified the expression of these target proteins, which were in accord with the qPCR and immunofluorescence results (n = 3). *p < 0.05 vs. Control.

**Figure 4:**
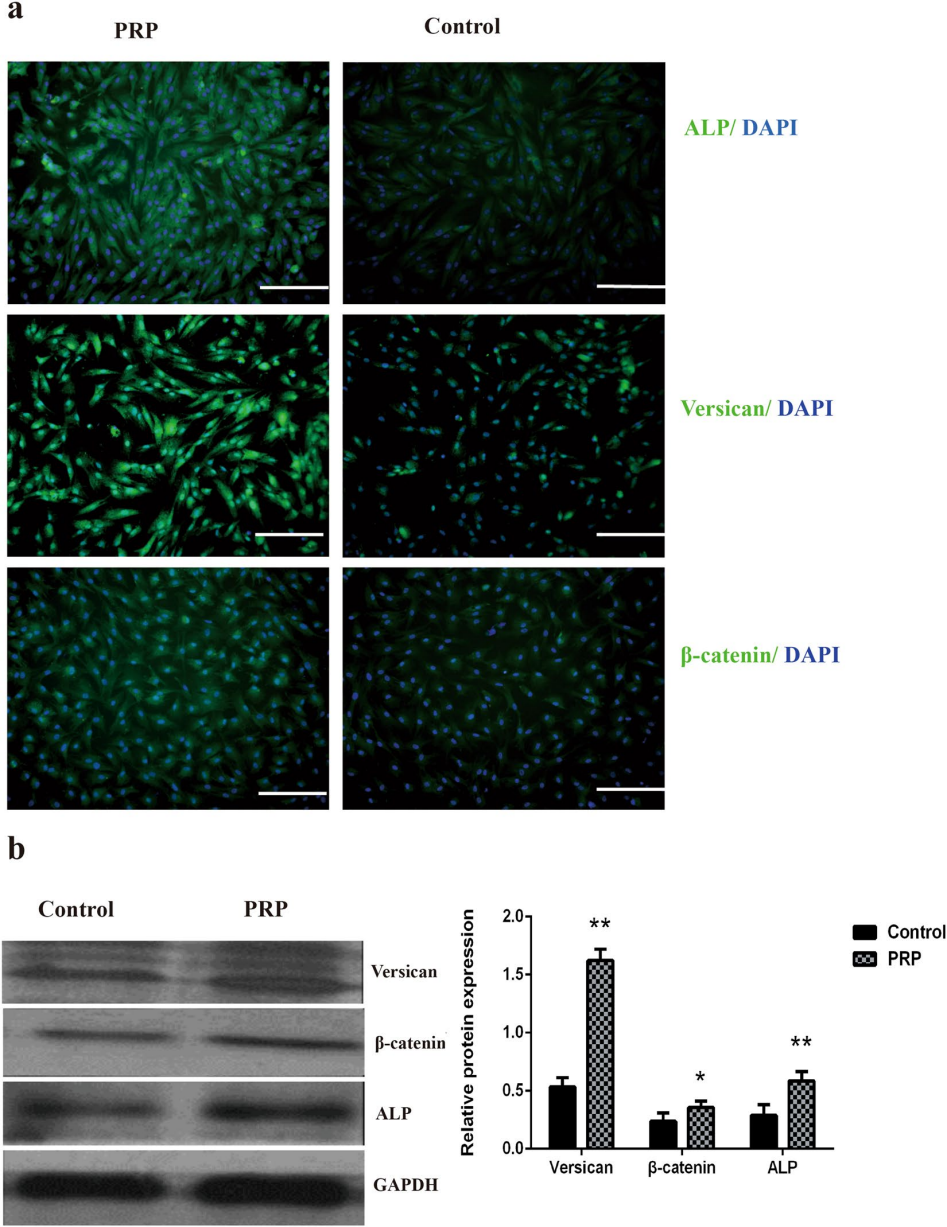
DPC marker proteins of human were assayed by immunofluorescence staining and immunoblotting. (**a**) Immunofluorescent images show that compared with controls, PRP-treated DPCs exhibited greater expression. Scale bars = 200 μm. (**b**) Western blot analysis quantified the expression of these target proteins, which were in accord with the qPCR and immunofluorescence results (n = 3). *p < 0.05 vs. Control.

**Figure 5:**
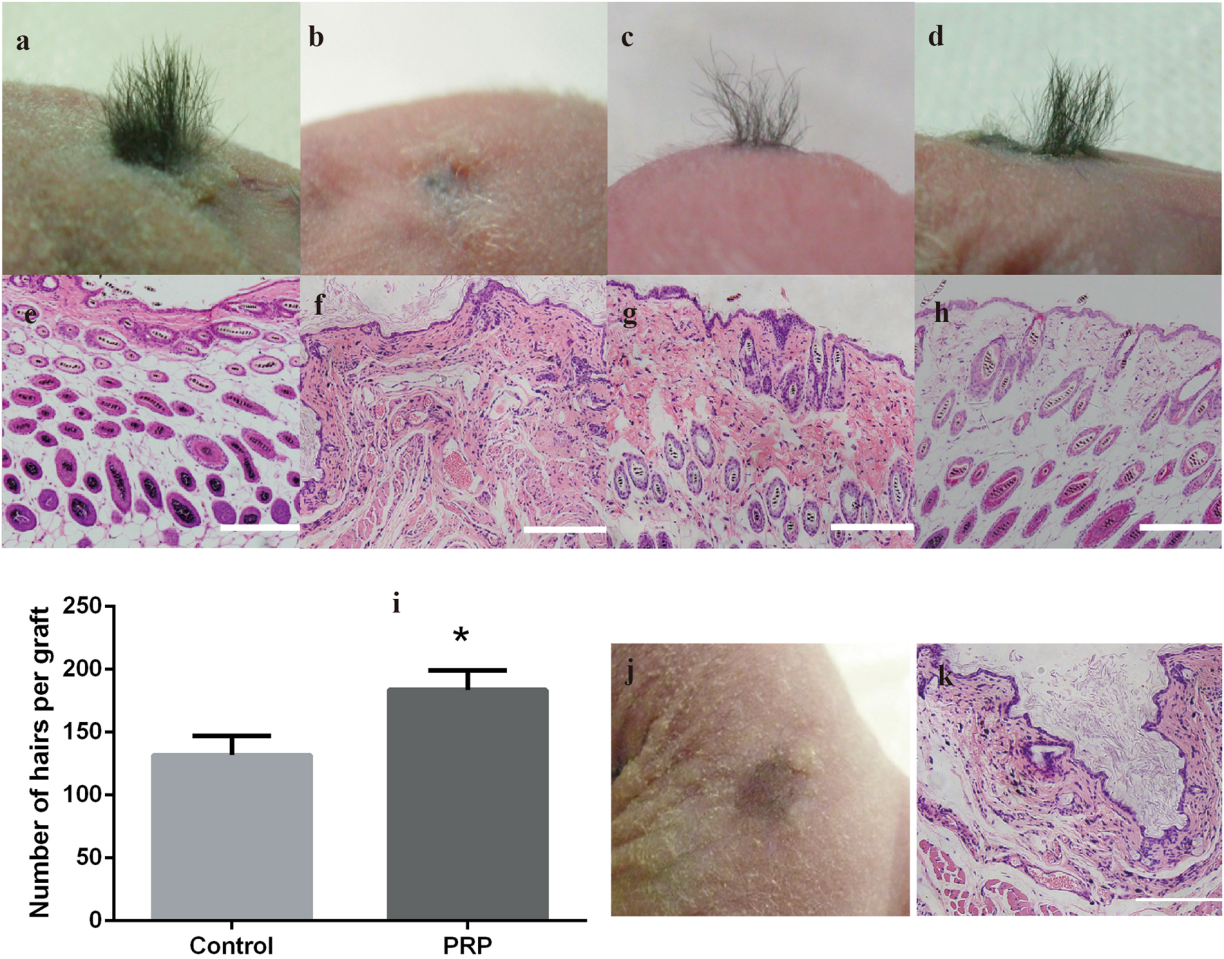
*In vivo* hair follicle reconstitution. The results of the mini-chamber assay showed that the positive control induced hair follicles (**a**), and the negative control did not (**b**), DPCs cultured in general medium induced sparse hair follicles (**c**). DPCs treated with 5% PRP induced more hair follicles (**d**). Corresponding haematoxylin and eosin-stained sections of each groups (**e**–**h**). Scale bars = 200 μm. The number of hairs induced by DPCs treated with 5% PRP was significantly higher than that induced by DPCs treated with culture medium alone (**i**). *P < 0.05 vs. Control. Cultured human DPCs engrafted with freshly isolated neonatal foreskin epidermal cells, no hair induction was observed by gross (**j**), and no hair follicle was observed within the skin of nude mice by histological examination (**k**).

The effects of activated PRP concentration on mouse DPCs are shown in Fig. 1a. The proliferation of mouse DPCs peaked on day 5 of culture in the presence of 5% activated PRP, and decreased in a dose-dependent manner in the presence of 10% or 15% activated PRP. As shown in Fig. 1b, the effect of different concentrations of activated PRP on proliferation of human DPCs and mouse DPCs was similar. The proliferation of human DPCs peaked on day 5 of culture in the presence of 5% activated PRP and decreased in a dose-dependent manner in the presence of 10% or 15% activated PRP. To determine the effect of activated PRP on the hair-inductive activity of DPCs, we assayed the expression of genes associated with hair induction such as *ALP* ^9, 32^, *β-catenin* ^33^ and *Versican* ^34^ by qPCR in DPCs cultured in general medium (control) and in medium supplemented with 5% activated PRP. As shown in Fig. 2a, ALP, β-catenin and Versican were expressed in mouse DPCs at significantly higher levels in medium with 5% PRP than in control medium. Similarly, the expression of these signature genes in human DPCs was higher in medium with 5% PRP than in control medium (Fig. 2b). The expression of ALP, β-catenin and Versican marker proteins were confirmed by immunofluorescence staining, and as with qPCR, expression of all three proteins was increased in the PRP group compared with controls in mouse DPCs (Fig. 3a). Significant differences in the expression of ALP, β-catenin and Versican by mouse DPCs in control and 5% activated PRP cultures were also seen in western blots (Fig. 3b and S1). Immunofluorescence staining and immunoblotting confirmed that the expression of ALP, β-catenin and Versican was also significantly up-regulated in human DPCs in the PRP group compared with controls (Fig. 4 and S2). Although, the expression of endogenous biological markers is assumed to indicate the hair-inductivity of DPCs *in vitro*, *in vivo* corroboration of these findings in *in vivo* models is needed. The mini-chamber assay was used to compare the hair-inductive activity of mouse DPCs at passage 3 cultured with neonatal mouse epidermal cells with or without 5% PRP. Positive controls always induced hair follicles (Fig. 5a and e), and negative controls did not induce any hair follicles (Fig. 5b and f). Although DPCs cultured in general medium (Fig. 5c and g) and DPCs cultured in medium with 5% PRP (Fig. 5d and h) both induced hair follicles, significantly more hair follicles formed in the presence of PRP than in the culture medium controls. The number of hairs induced by DPCs treated with 5% PRP was significantly greater than that induced by DPCs in control cultures (Fig. 5i). However, when cultured human DPCs and neonatal foreskin epidermal cells were transplanted in the mini-chamber assay, no hair follicle induction was observed, and the skin healed by wound contraction and re-epithelialization (Fig. 5j). Similarly, de novo hair follicle formation was not observed in histological sections of the skin at the transplantation site (Fig. 5k).

### Hair follicle reconstitution in PRP scaffolds

**Figure 6:**
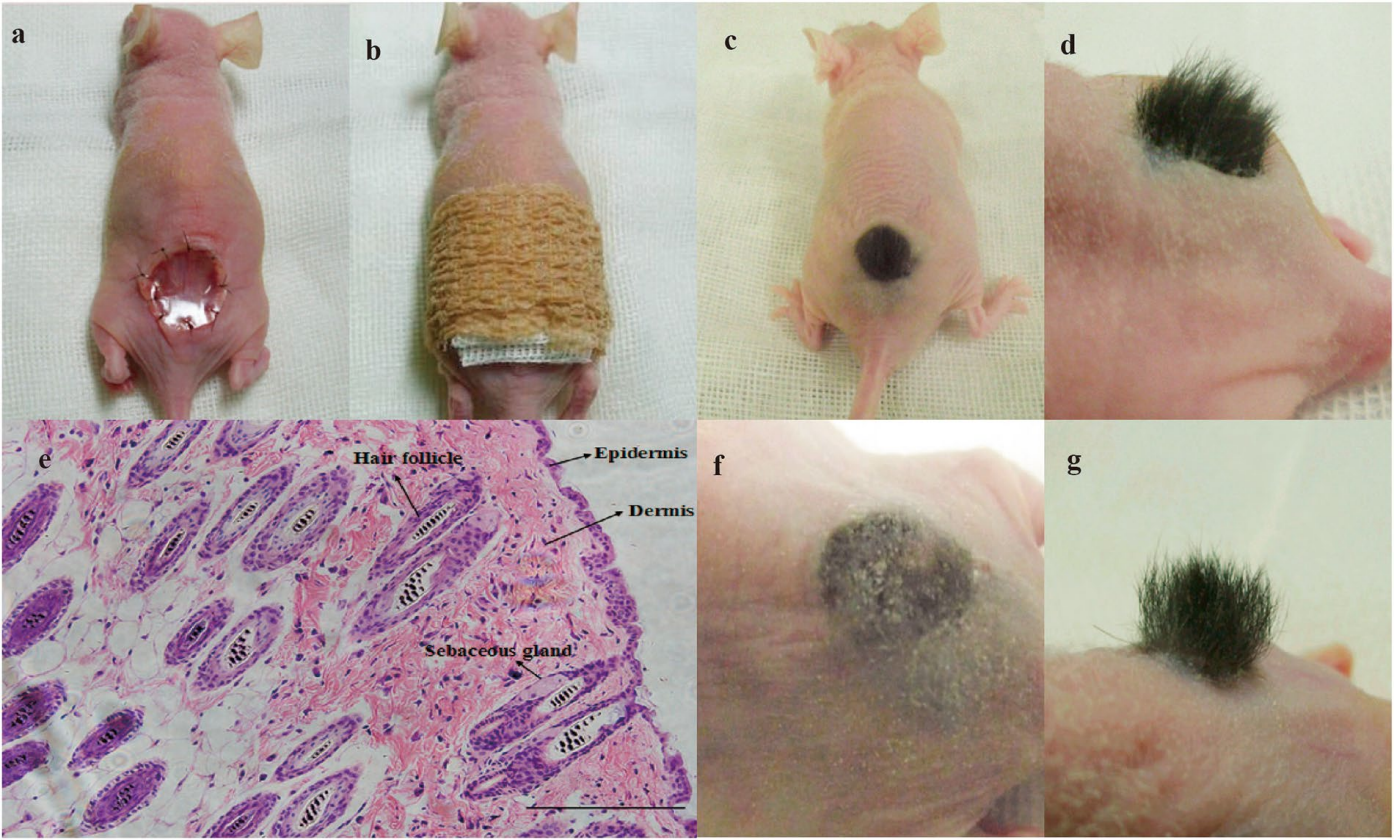
Graft appearance and results. (**a**) Scaffold and cells were positioned on the host. Interrupted sutures of the skin and a silicon membrane secure the graft in place. (**b**) Gauze and a tight elastic wrapping were used to dress the wound. (**c**,**d**) Dense black hairs were observed at the graft site 4 weeks after grafting. (**e**) Normal layers of skin regrew. Haematoxylin and eosin; scale bar = 200 μm. Reconstituted hair regrew after plucking: (**f**) Graft after hair was plucked; (**g**) Reconstituted hair regrew 4 weeks after plucking.

To determine whether PRP could function as a bioactive scaffold capable of endogenous growth factor release for hair follicle reconstitution, PRP gels containing mouse trichogenic cells were transplanted into nude mice. The appearance of a PRP gel graft with mouse trichogenic cells is shown in Fig. 6a and b. Hairs could be seen by the naked eye at grafted sites as early as 15–16 days after placement. The hairs were dense, arranged on a plane, grew evenly, and had a cosmetically acceptable appearance (Fig. 6c and d). Histological observation showed normal layers of skin at the grafted sites, including regenerated epidermis, dermis, hair follicles, and sebaceous glands (Fig. 6e). To evaluate regenerative ability, hairs that formed at the grafted sites were plucked with forceps. Hair fibres re-emerged from the skin and were visible within 2 weeks. The hairs continued to grow and reached a normal length in about 4 weeks (Fig. 6f and g). However, when PRP gels containing human DPCs and neonatal foreskin epidermal cells were transplanted into nude mice, hair follicles were not induced.

## Discussion

In this study, we evaluated the effects of PRP on cell growth of mouse and human DPCs and PRP as a bioactive scaffold for hair follicle tissue engineering. The viability and proliferation of mouse and human DPCs were stimulated by a low concentration of activated PRP; the addition of 5% activated PRP to the culture medium maximally promoted cell proliferation. Higher concentrations decreased of cell proliferation. The relatively high concentration of the mitogens PDGF-AB and FGF2 in activated PRP might account for the effect on proliferation.

Activated PRP is mitogenic for a variety of cell types, such as human adipose-derived stem cells, human dermal fibroblasts, rat calvarial bone cells, rat bone marrow-derived stem cells^35–38^. However, few data are available on its effect on DPCs. Li *et al*.^39^ reported that activated 5% PRP increased the proliferation of DP cells and protected them from apoptosis, but higher concentrations did not increase. Our results are consistent with those findings, and the necessity of reducing the PRP concentration to a level suitable for enhancing proliferation and hair follicle regeneration by DPCs.

Although 5% activated PRP maximally enhance mouse and human DPC proliferation maximally, the question remains as to whether it also enhanced hair-inductive activity. The addition of 5% activated PRP to the culture medium enhanced the hair-inductivity of mouse and human DPCs as measured by qPCR and immunofluorescence. Western blots confirmed the expression of these marker proteins. The mini-chamber assay showed that mature follicles were induced by cultured mouse DPCs and freshly isolated neonatal epidermis. Abundant hairs were observed with DPCs treated with PRP but not with untreated DPCs. Taken together, the data support the competency of 5% activated PRP to enhance the hair-inductive properties of mouse and human DPCs *in vitro* and mouse DPCs in an *in vivo* mini-chamber assay. The secretion of various growth factors after PRP was activated could explain this effect. FGF2 and PDGF have been shown to promote the hair-inductivity of DPCs^28–30^. Cultured mouse DPCs engrafted with freshly isolated neonatal mouse epidermal cells can reconstitute hair follicles. However, cultured human DPCs engrafted with freshly isolated neonatal foreskin epidermal cells could not. This might result from species-specific differences. Human scalp DPCs and of rodent vibrissal DPCs differ in their ability to aggregate both *in vitro* and *in vivo*. Cultured rat DPCs can self-aggregate to formed a papilla-like clump after subdermal injection, but spontaneous aggregation of cultured human DPCs injected into skin has not been observed^40, 41^. It has been shown that very rapid and extensive changes in molecular signature are associated with the transition of human DPCs from a three-dimensional to a two-dimensional environment, and that DPCs tend to lose their trichogenic ability when grown as traditional two-dimensional monolayers^42, 43^. Higgen *et al*. showed that the hair-inductive capability of human DPCs can be partially restored by growth in three-dimensional spheroid cultures, and that this partial restoration by itself is sufficient to initiate hair-follicle neogenesis^43^. In our study, 5% activated PRP enhanced the hair-inductive properties of human DPCs *in vitro*, but DPCs cannot induce hair follicles *in vivo*. One explanation is that the inductive ability of DPCs is not strong enough to initiate hair follicle neogenesis *in vivo*. Many studies suggest that for complete hair follicle morphogenesis, restoring the full functionality of cultured human DPCs to induce follicles, requires external paracrine signals in addition to repopulating DPCs into a three-dimensional environment. We hypothesize that a combination of 5% activated PRP culture with spheroid formation will lead to improved hair follicle induction, and may confer full inductivity to cultured human DPCs. In our study, cultured human DPCs engrafted with freshly isolated neonatal foreskin epidermal cells could not reconstitute hair follicles in the mini-chamber assay. We previously reported that cultured human DPCs engrafted with freshly isolated neonatal mouse epidermal cells also could not reconstitute hair follicles^44^. However, other investigators have induced chimeric human/rodent hair follicles in recipient rodent tissues^45, 46^. This could be the result of using different animal models for hair reconstitution. Although our data strongly suggest that 5% activated PRP enhanced the proliferative and hair-inductive properties of DPCs, this study was not without limitations. The underlying mechanism of PRP promotion of DPCs hair-inductivity remains the target of ongoing research. Moreover, we cannot say for certain that PDGF and FGF2 were the primary factors responsible for the proliferative and inductive effects seen without testing for effect reversal using anti-PDGF or anti-FGF2 antibodies.

PRP formed a three-dimensional gel after activation, and has been used as an autologous hydrogel containing growth factors, locally promoting tissue healing^47, 48^. Although PRP has seen increasing use in tissue repair and regeneration, its scaffolding potential has not been extensively investigated. Xie *et al*.^31^ reported that PRP matrixes had a three-dimensional mesh-like structure, and could act as a potential cell scaffold for cartilage engineering. Jalowiec *et al*.^49^ described the PRP gels, as stable viscoelastic hydrogels than can effectively deliver bioactive molecules and mesenchymal stem cells in various clinical applications. We found that a PRP gel scaffold could be used to encapsulate trichogenic cells, and platelet derived growth factors for hair follicle tissue engineering. We used PRP gel as a scaffold to form a large number of de novo hair follicles on a plane surface. One of the criteria to judge successful formation of engineered hair follicles is the ability of the follicle to cycle physiologically and to regenerate after plucking^50^. In our system, hairs regenerate after plucking and grew to a normal length. The PRP scaffold does have limitation, including low mechanical stiffness and rapid degradation *in vivo*. It has been reported that the mechanical properties of PRP- gels can be improved through additional cross linking or adjusting the fibrinogen content^31^. The addition of fibrinolytic inhibitors or some protease inhibitors might slow the degradation of PRP gels^31^.

In conclusion, this study showed that PRP contained approximately 7.1 times as many platelets as whole blood and a high concentration of PDGF-AB and FGF2 following PRP activation. The addition of 5% activated PRP to culture medium maximally promoted mouse and human DPC proliferation; higher concentrations did not. Activated 5% PRP was competent to enhance the hair-inductive properties of mouse and human DPCs *in vitro* and promoted mouse hair follicle formation *in vivo*. Moreover, PRP gel could be employed formed a three-dimensional scaffold capable of releasing endogenous growth factors for hair follicle tissue engineering. Although further work will be required to reconstitute fully functional human hair follicles, these findings represent a significant advance within the field of hair follicle tissue engineering.

## Methods

### Isolation of cells

All animals were provided by the Experimental Animal Centre at Southern Medical University (Guangzhou, China). and the experimental procedures were approved by the Institutional Animal Care and Use Committee. All procedures involving animals were performed in accordance with the relevant guidelines and regulations. Murine vibrissal DPCs were harvested from 4–5 week-old C57BL/6J mice as previously described^28^. Vibrissae pads were severed, the skin was inverted, and follicles were removed with fine forceps. The collagen capsules surrounding the follicles were dissected to expose the follicle base, and DPs were dissected using thin needles. Isolated DPs were transferred to cell cultures and grown in Dulbecco’s Modified Eagle’s Medium (DMEM, Gibco, Grand Island, NY, USA) supplemented with 10% (v/v) foetal bovine serum (Gibco) at 37 °C and 5% CO_2_. After being cultured for 5–7 days, DPCs were harvested with 0.25% trypsin–EDTA (Gibco), and transferred to fresh culture dishes. Passage 0 cells were used to test the effect of activated PRP on DPC proliferation. Mouse DPCs were cultured in DMEM with 10% foetal bovine serum, with or without activated PRP. The culture medium was changed every 3 days. Cells at passage 3 were used in the experiments described below.

Epidermal and dermal cells were isolated from C57BL/6J mice at natal day 0. Briefly, after digestion with 0.1% dispase (Invitrogen, Grand Island, NY, USA) at 4 °C overnight, epidermis was separated from the dermis. Then the epidermis was minced, and digested with 0.25% trypsin for 10 min at 37 °C to obtain freshly isolated epidermal cells. The dermis was minced, and digested with 0.2% collagenase I for 1 hour at 37 °C to obtain freshly isolated dermal cells.

Occipital scalp skin samples and neonatal foreskins were obtained from discarded tissue, after receiving ethical approval, and an institutional review board exemption from Southern Medical University. Informed consent was obtained from all subjects. All experimental protocols were approved by the Medical Ethical Committee of the Southern Medical University and all methods were performed in accordance with the relevant guidelines and regulations. Human DPCs were isolated and expanded as previously described^16^. Briefly, follicle bulbs were transected and dermal papilla were microdissected from the bulbs, transferred onto plastic dishes, and placed in culture medium. Passage 0 cells were used to test the effect of activated PRP on DPC proliferation. Human DPCs were cultured in DMEM with 10% foetal bovine serum, with or without activated PRP. Cells at passage three were used in the experiments described below. Foreskin epidermal cells were isolated from discarded neonatal foreskins. Briefly, skin was placed in 0.2% dispase overnight at 4 °C after which the epidermis and dermis were separated with forceps. The epidermis was minced and then digested in 0.25% trypsin for 15 min at 37 °C. The foreskin epidermal cells were disaggregated by passing through a 70 µm cell strainer to obtain freshly isolated cells.

### Preparation of activated PRP, growth factor assays, and formation of transplanted constructs

Blood samples were obtained from four healthy adult volunteers after receiving informed consent. All experimental protocols were approved by the Medical Ethical Committee of the Southern Medical University and all methods were performed in accordance with the relevant guidelines and regulations. PRP was enriched by a two-step centrifugation process as described previously^39^. Briefly, 40 ml whole blood was drawn from each volunteer in four tubes, each containing 1 ml 3.2% trisodium citrate (Sigma, St. Louis, MO, USA). The tubes were centrifuged at 660 *g* for 7 min. Subsequently, the yellow plasma and buffy coat (containing the platelets) were transferred to new tubes and centrifuged again at 2350 *g* for 5 min. A 1 ml volume of plasma and precipitated platelets was used as PRP. A 1:1 mixture of 0.5 M calcium chloride and thrombin (Sigma) was prepared in advance as an activator. A 10:1 mixture of PRP and activator was incubated for 10 minutes at room temperature and served as the activated PRP used in study procedures. Various concentrations of activated PRP were added to the culture medium, and after forming a flexible fibrin clot, the activated PRP was centrifuged at 16,600 *g* for 15 minutes and the supernatant was stored at −20 °C until use. The FGF2 and PDGF-AB concentration in the preparation of activated PRP were quantitatively assayed with a Quantikine enzyme-linked immunosorbent assay kit (Sigma). Growth factor concentrations were measured following the manufacturer’s instructions.

A few minutes after adding the activator, PRP preparations formed flexible fibrin clots that were used as a bioactive scaffold for cell seeding. To obtain PRP scaffolds and transplanted constructs, concentrated DPCs and epidermal cells were mixed with PRP just prior to the addition of the calcium chloride and thrombin activator. A few minutes later, a flexible construct comprising trichogenic cells, the bioscaffold, and growth factors had formed.

### Evaluation of the effect of activated PRP on DPCs *in vitro* and *in vivo*

To study the effect of activated PRP on proliferation of DPCs, passage 0 DPCs were seeded at a density of 4000 cells/well in 96-well culture plates. The cells were cultured in general culture medium supplemented with 0% (control), 1%, 5%, 10%, or 15% activated PRP for 1, 3, or 5 days. The proliferation of cultured DPCs was assayed with a Cell Counting Kit-8 (Sigma). The absorbance of culture media was measured at 450 nm using a multilabel counter (n = 3).

qPCR, immunofluorescence staining, and western blotting were used to study the hair-inductive property of activated PRP on DPCs *in vitro*. For qPCR, total RNA was extracted from DPCs using RNAiso Plus reagent (TaKaRa, Dalian, China). cDNA was synthesized from 2 μg of total RNA with a SYBR PrimeScript RT-PCR Kit (TaKaRa). qPCR was carried out using a SYBR PrimeScript RT-PCR Kit on a Stratagene MX3005 P qPCR system (Agilent Technologies, Santa Clara, CA, USA) following the manufacturer’s protocol. The fold change of each target gene was normalized to *GAPDH* mRNA. For immunofluorescence staining, cultured DPCs were fixed in 4% paraformaldehyde (Gibco) for 30 min at 4 °C. After rinsing three times with phosphate-buffered saline (Gibco), cells were permiabilised with 5% Triton X-100 (Sigma) for 10 min; 5% Bovine serum albumin (Gibco) was used for blocking for 30 min. The following primary antibodies were used: rabbit anti-ALP (1:100, Abcam, Cambridge, UK); rabbit anti-β-catenin and rabbit anti-Versican (1:200, Abcam). After incubation with primary antibodies overnight at 4 °C, cells were washed thoroughly with phosphate-buffered saline. A secondary antibody, Alexa Fluor488-conjugated goat anti-rabbit immunoglobulin G (1:200, Abcam) and DAPI (1:500, Sigma) were applied for 1 h at room temperature. Immunofluorescent images were recorded using a fluorescence microscopy system (IX71 FL, Olympus, Japan).

For western blotting, cell lysates were obtained using RIPA lysis buffer (Gibco), and 30 μg aliquots of total protein from each sample was subjected to sodium dodecyl sulphate polyacrylamide gel electrophoresis and transferred to a polyvinylidene fluoride membrane. After blocking, the blotted membranes were incubated with the following primary antibodies at 4 °C overnight: ALP (1:10000), β-catenin (1:5000); Versican (1:5000); and GAPDH (1:1000) monoclonal antibody. The blots were incubated with corresponding secondary antibodies (1:5000). The immune complexes were assayed with an enhanced chemiluminescence kit (Invitrogen) and analyst/PC densitometry software (Bio-Rad Laboratories, Hercules, CA, USA).

The mini-chamber assay was used to test the *in vivo* efficiency of hair induction by DPCs. The cell grafting procedure for hair follicle reconstitution using the mini-chamber assay was performed as described previously^44^. A mixture of cultured mouse DPCs and neonatal mouse epidermal cells was prepared for use in the mouse hair follicle reconstitution assay. Freshly isolated dermal and epidermal cells from C57BL/6 mice on natal day 0 were implanted as positive controls; only epidermal cells were implanted as negative controls. Cell mixtures (5 × 10^6^ epidermal cells and 5 × 10^6^ DPCs) in a total volume of 20 μl were transplanted into each mini-chamber. The mini-chambers were removed 1 week later. Hair reconstitution was evaluated by gross and histological observation. Four weeks later, the graft sites were harvested and paraffin sections were prepared. Histological sections of the grafts were stained with haematoxylin and eosin. Before processing, all hairs that had grown at the graft site were stripped using forceps and spread on paper for counting. A mixture of cultured human DPCs and neonatal foreskin epidermal cells was prepared for use in the human hair follicle reconstitution assay. Suspensions of 5 × 10^6^ epidermal cells and 5 × 10^6^ DPCs in a total volume of 20 μl were transplanted into each mini-chamber. The mini-chambers were removed 1 week later. Hair reconstitution was evaluated by gross and histological observation.

### PRP scaffold construct grafting in mice and evaluation of hair follicle reconstitution

Athymic nude mice were anesthetized, and the skin was sterilized with betadine solution. A 1 cm diameter wound was surgically created on the dorsum leaving the myolemma undamaged. PRP constructs containing either 1 × 10^7^ cultured mouse DPCs and 1 × 10^7^ neonatal mouse epidermal cells (mouse) or 1 × 10^7^ cultured human DPCs and 1 × 10^7^ neonatal foreskin epidermal cells (human) were transplanted separately onto the wound. A protective silicone membrane was sutured to the host skin. To ensure incorporation of the graft with the host skin, sterile dressings were applied. 1 week later, the sutures and protective silicone layer were removed. Hair reconstitution was evaluated by gross and histological observation. For histological observation, the skin of the grafted site was harvested, paraffin sections were prepared and stained with haematoxylin and eosin. To monitor hair regeneration after plucking, hairs were stripped from grafts using forceps and pictures were taken daily to record hair regeneration.

### Statistics

One-way analysis of variance was used for statistical analysis, with *p* < 0.05 considered statistically significant. Experimental data were expressed as the means ± standard deviation. All experiments were repeated at least three times.

## Electronic supplementary material

Supplementary Information
