# Supplementary material for: As a carrier–transporter for hair follicle reconstitution, platelet-rich plasma promotes proliferation and induction of mouse dermal papilla cells
Source: Sci Rep. 2017 Apr 25;7:1125. doi: 10.1038/s41598-017-01105-8 (PMC5430928; doi:10.1038/s41598-017-01105-8)
Supplement: Supplementary file 1 — Supplementary Information [file 41598_2017_1105_MOESM1_ESM.docx]

**As a carrier–transporter for hair follicle reconstitution, platelet-rich plasma promotes proliferation and induction of mouse dermal papilla cells**

Shun-E Xiao#, Yong Miao#, Jin Wang, Wei Jiang, Zhe-Xiang Fan, Xiao-Min Liu and Zhi-Qi Hu*

Department of Plastic and Aesthetic Surgery, Nan Fang Hospital, Southern Medical University, Guangzhou, Guangdong, 510515, China.

Correspondence to: Zhi-Qi Hu, 1838 North Guangzhou AV, Guangzhou 510515, China.

Tel: 0086-20-61641861; fax: 0086-20-87743734; E-mail: [huzhiqidr@163.com](mailto:huzhiqidr@163.com).

#These authors contributed equally to this study.


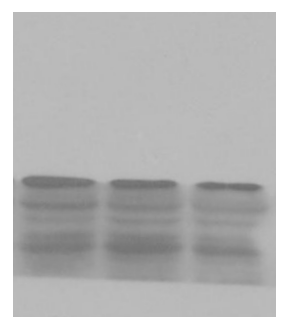

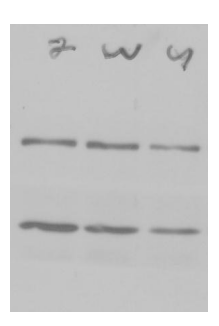


Versican β-catenin


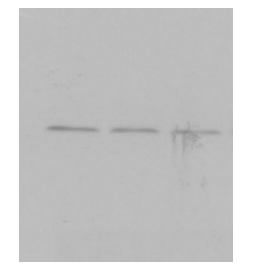

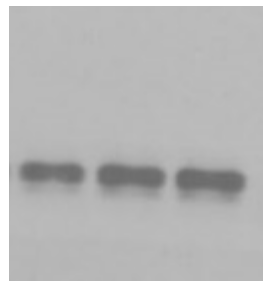


ALP GAPDH

Figure S1. Full-length blots of Figure 3.


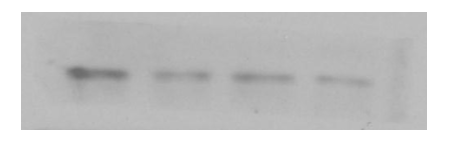


ALP


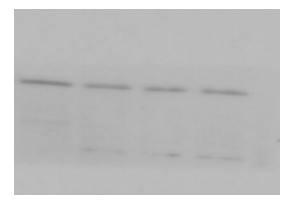


β-catenin


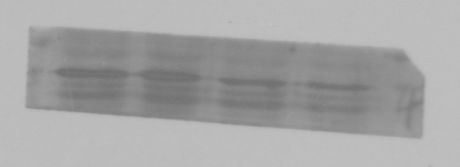


Versican


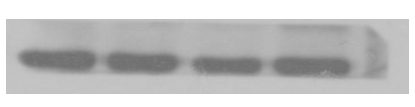


GAPDH

Figure S2. Full-length blots of Figure 4.
